# Supplementary material for: Target Recognition– and HCR Amplification–Induced In Situ Electrochemical Signal Probe Synthesis Strategy for Trace ctDNA Analysis
Source: Biosensors (Basel). 2022 Nov 8;12(11):989. doi: 10.3390/bios12110989 (PMC9688549; doi:10.3390/bios12110989)
Supplement: Supplementary file 1 [file biosensors-12-00989-s001.zip › biosensors-1965589-supplementary.pdf]

*Supporting information*

# Target Recognition– and HCR Amplification–Induced In Situ Electrochemical Signal Probe Synthesis Strategy for Trace ctDNA Analysis

**Aiting Cai [1], Luxia Yang [1], Xiaoxia Kang [1], Jinxia Liu [1,*], Feng Wang [1,2,*], Haiwei Ji [1], Qi Wang [1], Mingmin Wu [1], Guo Li [1], Xiaobo Zhou [1], Yuling Qin [1,*], and Li Wu [1,*]**

[1] Nantong University, No.9 Seyuan Road, Nantong, Jiangsu 226019, China
[2] Department of Laboratory Medicine, Affiliated Hospital of Nantong University, Medical School of Nantong University, Nantong 226001, China
* Correspondence: ljx1990@ntu.edu.cn (J.L.); richardwangf@163.com (F.W.); ylqin@ntu.edu.cn (Y.Q.); wuli8686@ntu.edu.cn (L.W.)

**Electrochemical characterizations of proposed biosensor**

As shown in Figure S1a, the bare gold electrode presented a sharp symmetrical redox peak, implying the great electron transfer rate (curve 1). The peak current decreased once the electrode was modified with capture probe, which can be attributed to the repulsion interaction between negatively charged DNA and $[Fe\ (CN)_6]^{3-/4-}$ (curve 2). MCH modification further led to the reduction of current because dense self-assembly layers impeded electronic transfer on the surface (curve 3). With the introduction of the target (curve 4) and initiator (curve 5), the peak current decreased further. Then, when hairpin H1 and H2 were introduced, HCR reaction occurred and produced a large number of double-stranded DNA molecules with negatively charged. The current further decreased (curve 6). Both the shift of potential and decrease of current in CV results confirmed the success of nucleic acid modification. The EIS characterization agreed with the CV results (Figure S1b), confirming the successful development of the electrochemical sensor again. The Nyquist plots of EIS consisted with the linear segment and the semicircle diameter. The former represented the lower-frequency region and the latter represented the higher-frequency region which stood for the electron-transfer resistance (Ret). With the continuous immobilization of the capture probe (curve b, 66.7 Ω), MCH (curve c, 147 Ω), target (curve d, 700 Ω), initiator (curve e, 1.71k Ω), hairpin H1 and H2 (curve f, 2.08k Ω) in the Au electrode (curve a, 45.1 Ω), the Ret values increased continuously which can be explained by the fact that the newly generated negatively charged DNA duplexes had a strong hindering effect on electron transfer. The above results proved that the construction of the EC-HCR sensor was successful.

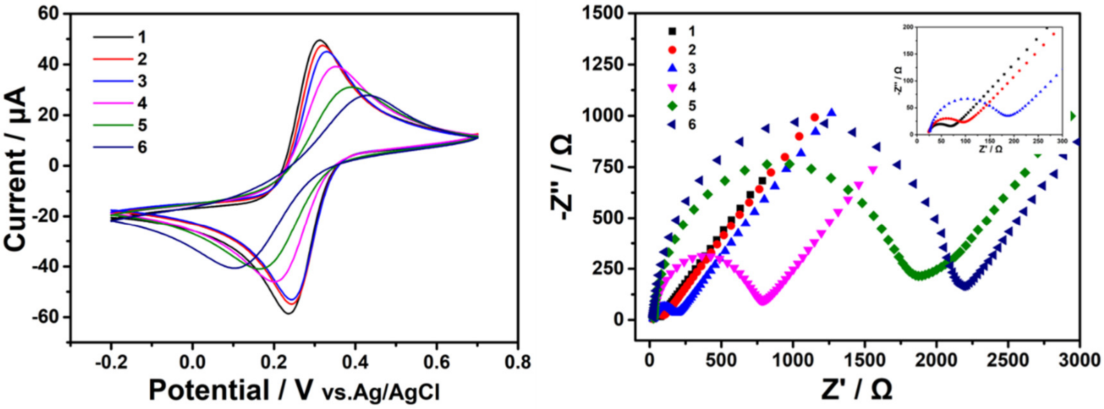


(a) (b)

**Figure S1. (a)** CV of different nucleic acids modified electrode in 10 mM $K_3[Fe(CN)_6]$ containing 1 M KCl. **(b)** EIS characterization of different nucleic acids modified electrode in 10 mM $K_3[Fe(CN)_6]/K_4[Fe(CN)_6]$ containing 1 M KCl. (1) bare Au electrode, (2) Capture probe/Au electrode, (3) MCH/Capture probe/Au electrode, (4) Target/MCH/Capture probe/Au electrode, (5) Initiator/Target/MCH/Capture probe/Au electrode and (6) Hairpin H1 and H2 /Initiator/Target/MCH/Capture probe/Au electrode.

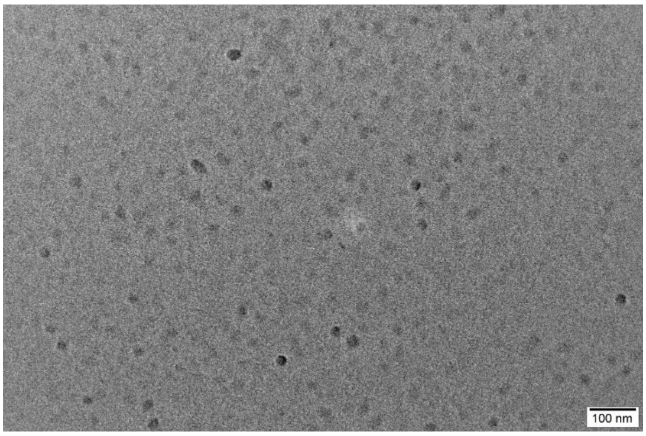


**Figure S2.** TEM image of DNA metallization product of silver nanoparticles.

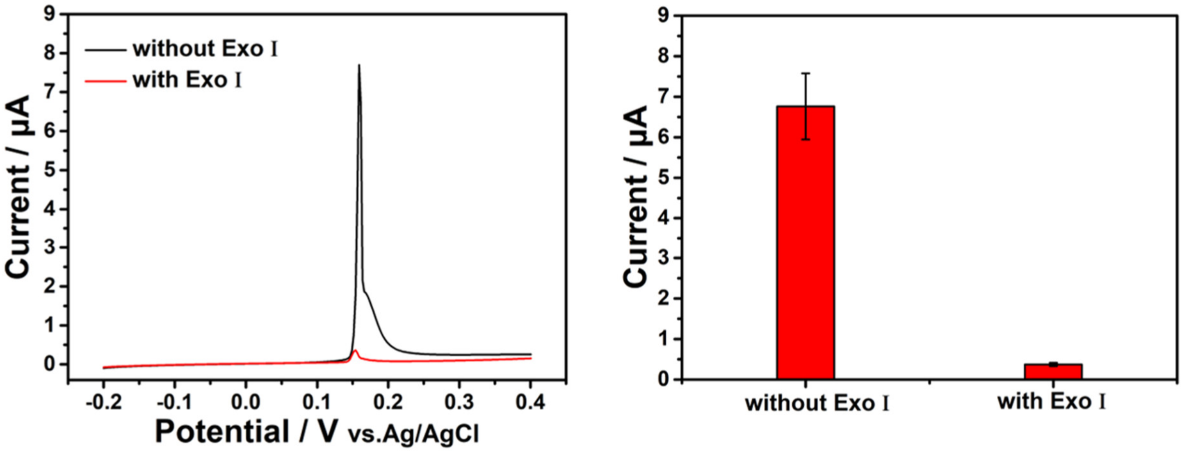


**Figure S3. (a)** The voltammetric responses of the samples without (black line) and with (red line) the treatment of Exo I in aqueous solution with 0.1 M $NaNO_3$ and 0.1 M NaCl. The electrode was modified with the target (1 aM), MCH, and capture probe. Scan rate：0.01 V/s. **(b)** The contrasts of multiple CV peak current without and with the function of Exo I. Each one was repeated 3 times.

**Optimization of the detection conditions**

In order to guarantee high hybridization efficiency, we further optimized the capture probe density and spatial arrangement. Low concentration of modified capture probes reduces the target binding sites, that is to say, affecting the efficiency of target recognition. However, the dense nucleic acid probe layer also has an adverse effect on sensing performance as it increases the steric hindrance of the probe recognition. Therefore, the density of capture probes modified on the electrode surface needs to be optimized. When the concentration of the target was set as 2 μM, the peak current of silver solid transition revealed that the optimal concentration for the capture probe was 0.05 μM (Figure S4a). The chronocoulometric intercept difference ($Q_{total}$ and $Q_{dl}$) represents the excess charge of the surface, which can be converted into the density of DNA. According to the calculating equation [1], the density of capture probe on the electrode interface was $6.08\times10^{12}$ molecules $cm^{-2}$ (Figure S5, Calculation process see supporting information). The time that the capture probe incubated with the target was also crucial to the experiment, no matter it is too short or too long, both do harm to sensor detection. As shown in Figure S4b, 1.5 h is the most suitable time for incubation obviously. The initiator played a connecting role in linking the target and hairpin H1 and related to the triggering of HCR. The oxidation current reached the maximum at the concentration of 0.5 μM (Figure S4c). The concentration and incubation time of hairpin H1 and H2 were also critical for the HCR. As illustrated in Figure S4d, the current intensity increased with the increasing concentration of H1 and H2 and reached a plateau at 1.5 μM, implying a suitable concentration for downstream assays. Figure S4e shows that the electrochemical signals reached saturation when the incubation time of HCR was prolonged to 1.5 h.

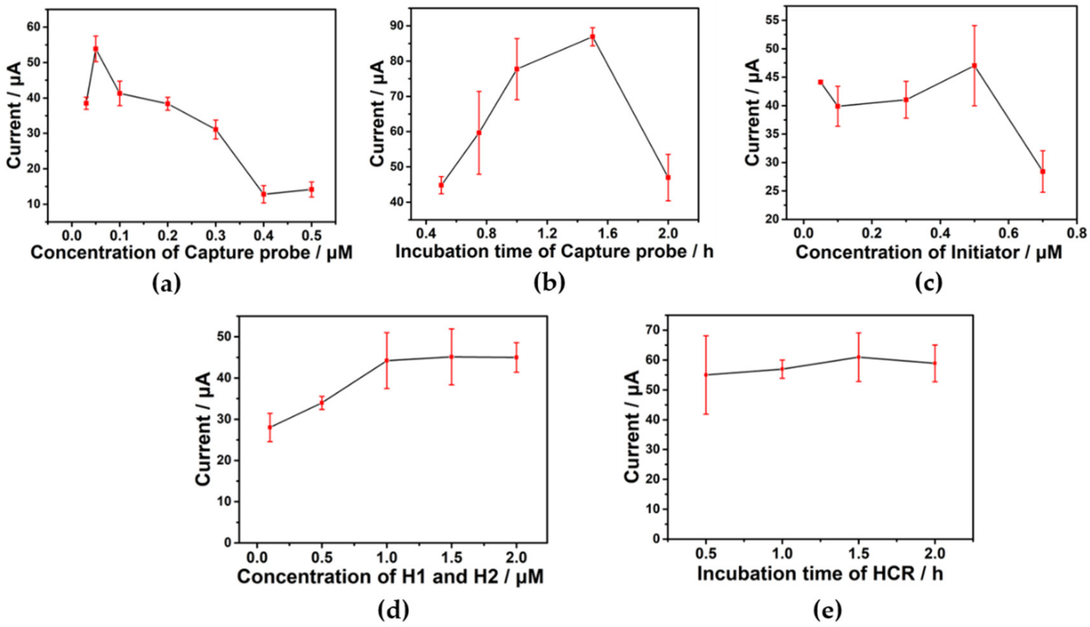


**Figure S4.** **(a)** Optimization of capture probe immobilization concentration. **(b)** Optimization of capture probe incubation time. **(c)** Optimization of initiator concentration. **(d)** Optimization of hairpin H1 and H2 concentration. **(e)** Optimization of HCR incubation time.

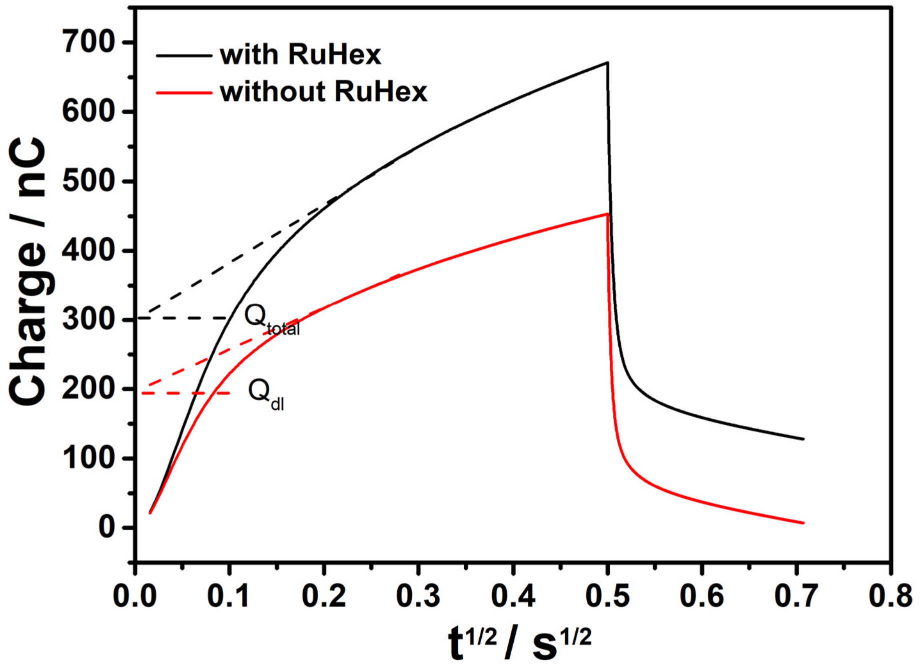


**Figure S5.** Chronocoulometric plot of MCH/capture probe immobilized on gold electrode in the absence (red line) and presence (black line) of 50 μM RuHex. The concentration of capture probe: 0.05 μM.

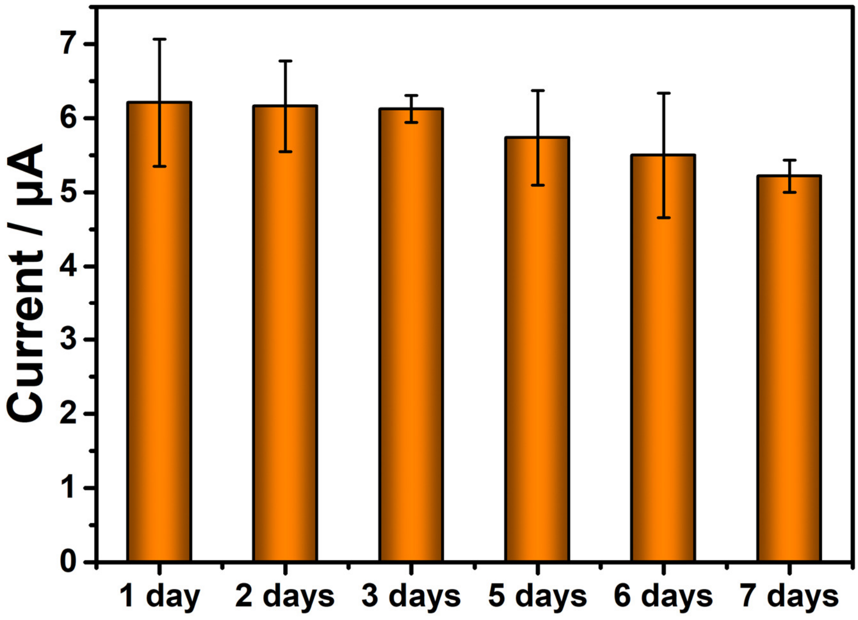


**Figure S6.** Stability of electrochemical sensors (Target: 1 nM).

**Calculation process:**

For Chronocoulometric plot: Chronocoulometric experiments can be used to perform quantitative calculations of DNA density on the electrode. The DNA surface density can be converted from the excess surface charge. The integrated current or charge Q as a function of time t in the chronocoulometric experiment is given by the integral Cottrell expression,

$$Q = \frac{2nFAD_0^{1/2}C_0^*}{\pi^{1/2}}t^{1/2} + Q_{dl} + nFA\Gamma_0$$

The chronocoulometric intercept at time=0 is the sum of the double layer charge and the surface excess charge. According to Faraday's law, the reduced charge of RuHex can be expressed as

$$\Gamma_{Ru} = \frac{Q_{ds} - Q_{dl}}{nFA}$$

The surface excess charge is converted to the surface density of DNA probe under saturated conditions,

$$\Gamma_{DNA} = \Gamma_{Ru}\left(z/m\right)\left(N_A\right)$$

After the final conversion, we can get the formula

$\Gamma_{DNA}$ = 【$(Q_{ds}$-$Q_{dl})/(nFA)$】$(z/m)(N_A)$

Where n is the number of electrons per molecule reduced. Faraday's constant (F) is a physical constant representing the charge carried per mole of electrons and is generally calculated by taking a value of 96485 C/mol. A is the area of a single electrode. z is the charge of the redox molecule and RuHex is 3. m is the number of bases in the probe DNA. Avogadro number ($N_A$) is the thermal constant and is generally calculated by taking the value $6.022\times10^{23}$.

For range and limit of detection: The peak currents of concentration of target ranging 0.5 fM to 10 pM are shown in the table below. The linear correlation curve is described as

y=1.0399 x+0.5314. The slope (S) is 1.0399. When the test solution is blank buffer, the peak currents are 0.1422 μA, 0.1463 μA and 0.1466 μA. Standard deviation (N) of blank multiple determinations is 0.0025. With the formula LOD=3N/S, limit of detection (LOD) is 7aM.

| The concentration of target | Take the log of the concentration of target | The peak current (μA) | | | | |
|---|---|---|---|---|---|---|
| **0.5 fM** | **-0.3** | **0.1985** | **0.235** | 0.2161 | | |
| 1 fM | 0 | 0.5188 | 0.7056 | 0.6555 | | |
| 10 fM | 1 | 1.0809 | 0.957 | 2.0242 | | |
| 0.1 pM=100 fM | 2 | 2.4274 | 2.8165 | 2.6028 | | |
| 1 pM | 3 | 4.0768 | 3.1299 | 3.0347 | | |
| 10 pM | 4 | 5.8014 | 3.6246 | 3.4851 | 4.5911 | 5.7617 |

**Table S1.** Comparison with previous literature of similar tests.

| Detection method | Analysis method | The volume of target used for the analysis | Detection range | LOD | References |
|---|---|---|---|---|---|
| EC | DPV | 10 μL | 1 fM-10 nM | 0.29 fM | [2] |
| SERS | Raman spectra | - | 0.1 fM-10 nM | 0.12 fM | [3] |
| ECL | ECL intensity | - | 1 fM-1 nM | 0.8 fM | [4] |
| EC | DPV | - | 10 fM-10 nM | 1.6 fM | [5] |
| EC | DPV | 10 μL | 100 fM-100 nM | 7.65 fM | [6] |
| EC | DPV | - | 0.01 pM-500 pM | 0.13 pM | [7] |
| EC | Amperometric i-t curves | 20 μL | 0.1 fM-10 nM | 36 aM | [8] |
| EC | DPV | 10 μL | 5 pM-0.5 nM | 3 pM | [9] |
| SERS | SERS spectra | - | 10 fM-1.0 nM | 0.3 fM | [10] |
| EC | SWV | - | 100 aM-1 nM | 48 aM | [11] |
| EC | CV | 6 μL | 0.5 fM-10 pM | 7 aM | This work |

*Square wave voltammograms (SWV), Differential Pulse Voltammetry (DPV), Cyclic Voltammetry (CV), Electrochemiluminescence (ECL), Electrochemical (EC), Surface-enhanced Raman scattering (SERS)

**Table S2.** Spiked recovery experiments.

| Spiked | Measured | Recovery | RSD |
|---|---|---|---|
| 0.5 fM | 0.51 fM | 102.0% | 4.3% |
| 1 fM | 1.06 fM | 106.0% | 6.5% |
| 10 fM | 9.57 fM | 95.7% | 3.8% |

The "Spiked" represented the ctDNA concentration added to the complex matrix solution.

The "Measured" represented the ctDNA concentration average value (three times) got by the current signal and the linear equation.

The "Recovery" was calculated by "Spiked" and "Measured".

The "RSD" was calculated by "Measured".

**Table S3.** All sequences involved in this work.

| Name | Sequence (5′-3′) |
|---|---|

| | |
|---|---|
| Capture probe | HS-$(CH_2)_6$ C TAC GCC ACT |
| Target 134A allele | GTT GGA GCT AGT GGC GTA G [12] |
| Initiator | AGC TCC AAC AGT CTA GGA TTC GGC GTG GGT TAA [13-15] |
| Hairpin H1 | CAA AGT AGT CTA GGA TTC GGC GTG TTA ACC CAC GCC GAA TCC TAG ACT [13-15] |
| Hairpin H2 | CAC GCC GAA TCC TAG ACT ACT TTG AGT CTA GGA TTC GGC GTG GGT TAA [13-15] |
| mismatch 1 | GT*A* GGA GCT AGT GGC GTA G [16] |
| mismatch 2 | GTT GGA GCT AGT GGC *C*TA G [16] |
| mismatch 3 | GTT GG*C A*CT AGT GGC GTA G [16] |
| mismatch 4 | GTT GGA GCT AGT G*TA* GTA G [16] |
| mismatch 5 | GT*A CC*A GCT AGT GGC GTA G [16] |
| mismatch 6 | GTT GGA GCT AGT GG*T AG*A G [16] |

**References**


1 Steel, A.B.; Herne, T.M.; Tarlov, M.J. Electrochemical quantitation of DNA immobilized on gold *Anal. Chem*. **1998**, *70*, 4670-4677.
2 Li, D.; Xu, Y.; Fan, L.; Shen, B.; Ding, X.; Yuan, R.; Li, X.; Chen, W. Target-driven rolling walker based electrochemical biosensor for ultrasensitive detection of circulating tumor DNA using doxorubicin@tetrahedron-au tags. *Biosens. Bioelectron*. **2020**, *148*, 111826.
3 Zhang, J.; Dong, Y.; Zhu, W.; Xie, D.; Zhao, Y.; Yang, D.; Li, M. Ultrasensitive detection of circulating tumor DNA of lung cancer via an enzymatically amplified SERS-based frequency shift assay. *ACS. Appl. Mater. Interfaces*. **2019**, *11*, 18145-18152,
4 Feng, Y.; Sun, F.; Wang, N.; Lei, J.; Ju, H. $Ru(bpy)_{(3)}^{(2+)}$ incorporated luminescent polymer dots: Double-enhanced electrochemiluminescence for detection of single-nucleotide polymorphism. *Anal. Chem*. **2017**, *89*, 7659-7666.
5 Jia, L.P.; Zhao, R.J.; Feng, Z.; Wang, M.Y.; Ma, R.N.; Jia, W.L.; Shang, L.; Zhang, W.; Xue, Q.W.; Wang, H.S. Ultrasensitive electrochemical detection of circulating tumor DNA by hollow polymeric nanospheres and dual enzyme assisted target amplification strategy. *Sensors and Actuators B: Chemical*. **2022**, *350*, 130849.
6 Guo, L.; Mu, Z.; Yan, B.; Wang, J.; Zhou, J.; Bai, L. A novel electrochemical biosensor for sensitive detection of non-small cell lung cancer ctDNA using NG-PEI-COFTAPB-TFPB as sensing platform and Fe-MOF for signal enhancement. *Sensors and Actuators B: Chemical*. **2022**, *350*, 130874.
7 Chen, M.; Wu, D.; Tu, S.; Yang, C.; Chen, D.; Xu,Y. CRISPR/Cas9 cleavage triggered ESDR for circulating tumor DNA detection based on a 3d graphene/ AuPtPd nanoflower biosensor. *Biosens. Bioelectron*. **2020**, *173*, 112821.
8 Chen, K.; Zhao, H.; Wang, Z.; Lan, M. A novel signal amplification label based on aupt alloy nanoparticles supported by high-active carbon for the electrochemical detection of circulating tumor DNA. *Anal. Chim. Acta*. **2021**, *1169*, 338628.
9 Huang, Y.; Tao, M.; Luo, S.; Yang, C.; Chen, D.; Xu, Y. A novel nest hybridization chain reaction based electrochemical assay for sensitive detection of circulating tumor DNA. *Anal. Chim. Acta*. **2020**, *1107*, 40-47.
10 Zhou, Q.; Zheng, J.; Qing, Z.; Zheng, M.; Yang, J.; Yang, S.; Ying, L.; Yang, R. Detection of circulating tumor DNA in human blood via DNA-mediated surface-enhanced raman spectroscopy of single-walled carbon nanotubes. *Anal. Chem*. **2016**, *88*, 4759-4765.
11 Chai, H.; Miao, P. Ultrasensitive assay of ctdna based on DNA triangular prism and three-way junction nanostructures. *Chin. Chem. Lett*. **2021**, *32*, 783-786.
12 Das, J.; Ivanov, I.; Montermini,; L. Rak, J.; Sargent, E. H.; Kelley, S.O. An electrochemical clamp assay for direct, rapid analysis of circulating nucleic acids in serum. *Nat Chem*. **2015**, *7*, 569-575
13 Xing, S.; Lu, Z.; uang, Q.; Li, H.; Wang, Y.; Lai, Y.; He, Y.; Deng, M.; Liu, W. An ultrasensitive hybridization chain reaction-amplified CRISPR-Cas 12a aptasensor for extracellular vesicle surface protein quantification. *Theranostics*. **2020**, *10*, 10262-10273

14 Zhou, J.; Fu, R.; Liu, H.; Liu, Y.; Wang, Y.; Jiao, B.; He, Y.; Tang, H. Integrating multiple hybridization chain reactions on gold nanoparticle and alkaline phosphatase-mediated in situ growth of gold nanobipyramids: An ultrasensitive and high color resolution colorimetric method to detect the meca gene of staphylococcus aureus. *J Hazard Mater*. **2021**, *418*
15 Wang, C.; Zhou, H.; Zhu, W.P.; Li, H.B.; Jiang, J.H.; Shen, G.L.; Yu, R.Q. Ultrasensitive electrochemical DNA detection based on dual amplification of circular strand-displacement polymerase reaction and hybridization chain reaction. *Biosens. Bioelectron*. **2013**, *47*
16 Chai H,; Tang Y,; Guo Z,; Miao, P. Ratiometric electrochemical switch for circulating tumor DNA through recycling activation of blocked DNAzymes. *Anal Chem*. **2022**, *94*, 2779-2784
